# Predicting gene function in a hierarchical context with an ensemble of classifiers

**DOI:** 10.1186/gb-2008-9-s1-s3

**Published:** 2008-06-27

**Authors:** Yuanfang Guan, Chad L Myers, David C Hess, Zafer Barutcuoglu, Amy A Caudy, Olga G Troyanskaya

**Affiliations:** 1 Department of Molecular Biology, Princeton University, Princeton, NJ 08544, USA; 2 Lewis-Sigler Institute for Integrative Genomics, Princeton University, Princeton, NJ 08544, USA; 3 Department of Computer Science, Princeton University, Princeton, NJ 08544, USA

## Abstract

**Background:**

The wide availability of genome-scale data for several organisms has stimulated interest in computational approaches to gene function prediction. Diverse machine learning methods have been applied to unicellular organisms with some success, but few have been extensively tested on higher level, multicellular organisms. A recent mouse function prediction project (MouseFunc) brought together nine bioinformatics teams applying a diverse array of methodologies to mount the first large-scale effort to predict gene function in the laboratory mouse.

**Results:**

In this paper, we describe our contribution to this project, an ensemble framework based on the support vector machine that integrates diverse datasets in the context of the Gene Ontology hierarchy. We carry out a detailed analysis of the performance of our ensemble and provide insights into which methods work best under a variety of prediction scenarios. In addition, we applied our method to *Saccharomyces cerevisiae *and have experimentally confirmed functions for a novel mitochondrial protein.

**Conclusion:**

Our method consistently performs among the top methods in the MouseFunc evaluation. Furthermore, it exhibits good classification performance across a variety of cellular processes and functions in both a multicellular organism and a unicellular organism, indicating its ability to discover novel biology in diverse settings.

## Background

An important challenge in the post-sequence era of modern biology is determining the functional role of all proteins in the cell. With the recent invention of several large-scale experimental methods, we have begun to accumulate a wealth of functional genomic data to help address this challenge, including expression and protein-protein interaction data, and phenotype and phylogenetic profiles. These large datasets have fuelled an interest in computational approaches to gene function prediction, which promise to harness the information present in these large collections of data to automatically derive accurate gene annotations [1-7].

Although a variety of computational approaches have been proposed for predicting gene function, most of them have been developed and applied in unicellular organisms such as *Saccharomyces cerevisiae*. Applying these methods to higher, multicellular organisms is non-trivial because of their intrinsic complexity, including different development stages, tissue types and physiological functions. However, we perhaps have the most to gain from computational annotation efforts based on diverse data, as our knowledge about the role of individual proteins in these systems is largely incomplete. In fact, although organisms such as the laboratory mouse tend to be of great scientific importance, the majority of their proteins are annotated electronically based on only a single data source (the IEA [inferred from electronic annotation] evidence code) [8]. Many sources of genome-scale data are available for most genes and, thus, functional prediction from multiple data sources for multicellular organisms is an important open problem whose solution is critical to functional genomics.

To explore the development of gene function prediction methods for multicellular organisms, several groups recently participated in an organized function prediction project for the laboratory mouse, MouseFunc [9]. Several state-of-the-art machine learning methods were applied, including support vector machines (SVMs), Bayesian networks, decision trees and random forests. Here, we describe our contribution to this effort, an ensemble classifier approach that is based on the SVM and integrates information in the context of the Gene Ontology (GO) hierarchy.

Our approach is motivated by three key aspects of the gene function prediction problem. First, we hope to accurately annotate function for a broad range of biological processes, molecular functions and cellular components. Given this diversity, it is unlikely that one learning model will perform the best in all possible contexts, thus motivating our choice of an ensemble of complementary approaches. Second, established knowledge of gene function (that is, the gold standard for learning) is organized in the hierarchical structure of the GO, which can be leveraged to improve overall prediction accuracy. Finally, the available genomic data (for example, gene expression and protein-protein interaction data) are heterogeneous both in terms of the functional information they capture and in their inherent structure; a model for learning must be flexible enough to accommodate these differences and leverage functional diversity within the data.

**Figure 1:**
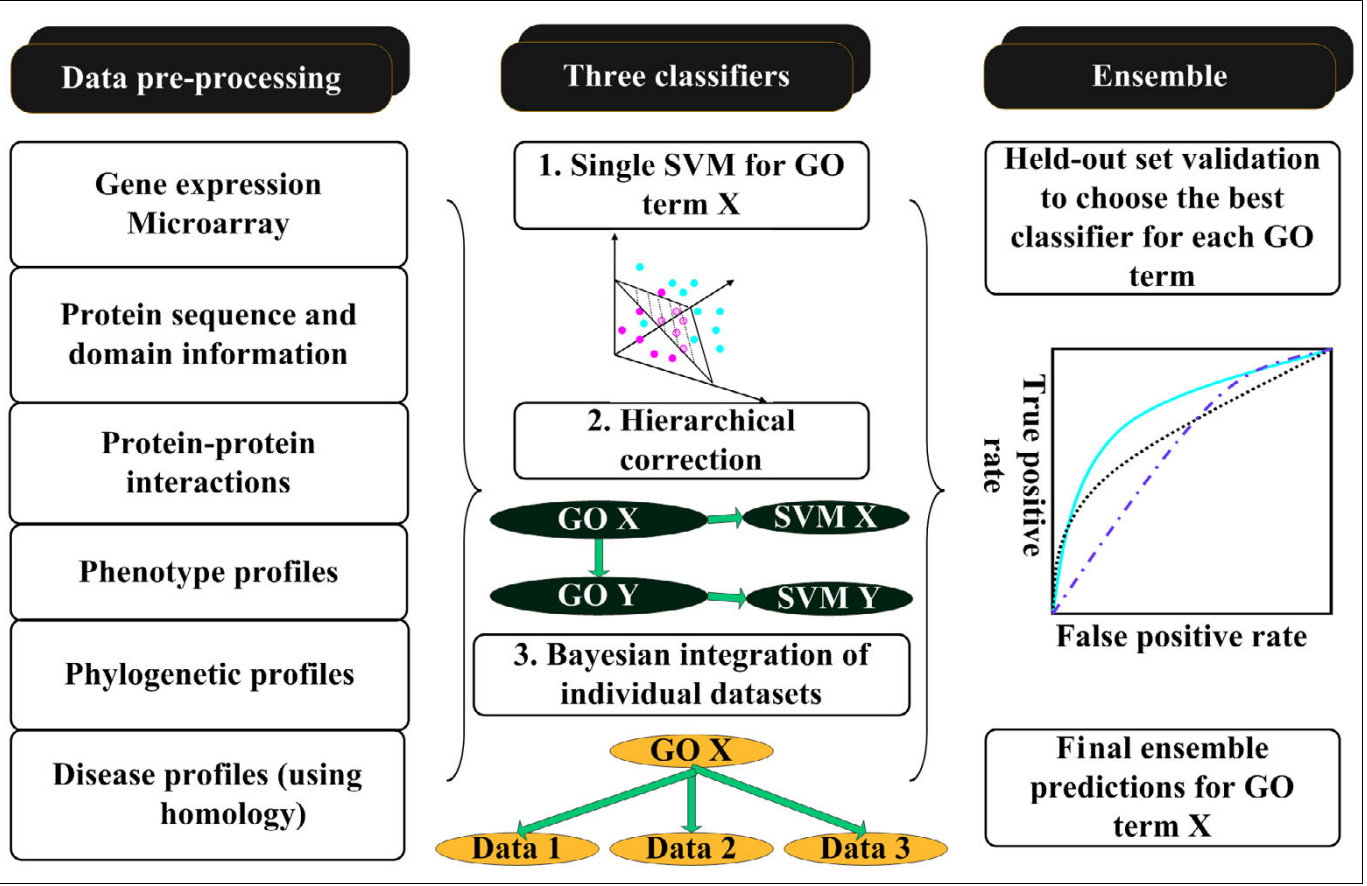
An ensemble framework based on the SVM that integrates diverse datasets in the context of GO hierarchy. After pre-processing the data, we developed an approach that consists of an ensemble of three different classifiers: 1, a single SVM classifier for each GO term was trained on combined data; 2, single SVM classifiers were combined through Bayesian networks to correct their predictions based on the hierarchical relationship between GO terms in the GO directed acyclic graph; and 3, a naïve Bayes classifier was built for each GO term to directly integrate the results of single-dataset SVM classifiers. The bootstrap held-out values on the training set were used to characterize each classifier's performance, and the ensemble prediction was formed by selecting the best performing classifier on each GO term. GO, Gene Ontology; SVM, support vector machine.

Our ensemble classifier is based on the SVM and leverages these characteristics unique to the gene function prediction setting (Figure 1). We illustrate that a combination of complementary strategies often outperforms a single SVM classifier and, furthermore, demonstrates consistently strong performance relative to competing groups in the MouseFunc submissions [9]. In the sections that follow, we provide an analysis of prediction performance and insights into when and why our ensemble framework performs well. Finally, we also demonstrate that our method can confidently predict novel biology with experimental confirmation of predictions in *S. cerevisiae*.

## Results

Our approach consists of an ensemble of three different classifiers, all based on the SVM: a single SVM, single kernel classifier; a hierarchically corrected combination of SVM classifiers; and a naïve Bayes combination of single dataset SVM classifiers (Figure 1). The basic process we applied was to train all three classifiers for each GO term, use out-of-bag bootstrap values to assess their performance, and finally assemble combined predictions based on classifier performance. We describe each of these approaches and their combination into the ensemble framework in detail in Materials and methods.

We applied this method in the MouseFunc GO prediction project [9]. This project involved prediction of a total of 1,726 biological process terms, 326 cellular component terms and 763 molecular function terms for laboratory mouse. As described in [9], methods were evaluated according to two different benchmarks. The first set included 1,718 genes whose annotations were held-out in advance, and the second set consisted of novel annotations, including 1,954 genes, added by Mouse Genome Informatics (MGI) in the approximately 8 months between dataset assembly and the prediction submission deadline. Here we focus our analysis on the latter set, which could be considered the ideal test case to determine how well our approach can predict completely new biology. Furthermore, to simplify the discussion, we have also restricted most of our analysis to the biological process GO terms. The trends discussed here are similar for the other types of terms (cellular component and molecular function) and the prediction of biological process terms is generally more difficult as shown by lower average areas under receiver operating characteristic (ROC) curves (AUCs; see Figure 2 in [9]).

**Figure 2:**
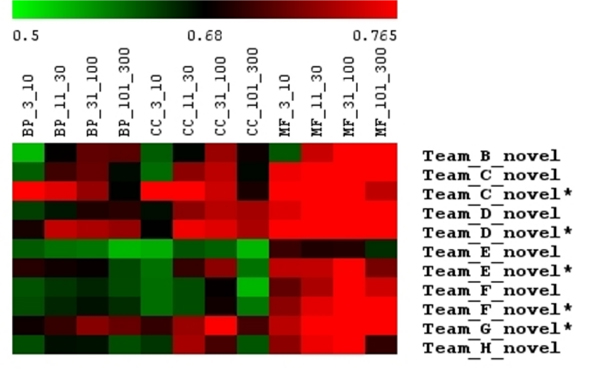
GO term size influences the performance of individual methods. GO term size appears to be a significant factor that influences the relative performance among groups (the performance of each group is shown by the average AUC values). Although there is no general trend common to all groups, individual groups demonstrate consistent trends regardless of what GO branch was tested. For example, groups B and D perform better on big GO terms. Group C performs better on small terms, and groups E to H perform well on intermediate-sized GO terms. Asterisks indicate second round submissions. AUC, area under receiver operating characteristic curve; BP, biological process; CC, cellular components; GO, Gene Ontology; MF, molecular functions.

### The ensemble method performs among the top prediction methods

Our ensemble approach performs well across a broad range of processes (GO terms), including both large and small terms from all GO branches. We measure an overall average AUC of 0.72, with an average precision of 0.13 at 20% recall. Of the 2,172 total terms across all three ontologies, there are 188 with better than 90% precision at 20% recall. Furthermore, our method always performs in the top three of the nine MouseFunc submissions in terms of the average AUC across all categories of terms. These categories were defined by the project organizers for each GO branch (biological process, cellular component, and molecular function) based on the number of annotations to each term [9]. Our method achieves the best average AUC for 3 of these 12 categories, the second best AUC for 6 of the 12, and the third best AUC for 3 of the 12 (Table 1 and Figure 2).

**Table 1:** Ranking of the performance of our group based on the mean AUCs

GO term size	Biological process	Cellular component	Molecular function
Test set ranking			
3-10	3	3	2
11-30	3	2	3
31-100	2	1	4
101-300	2	1	3
			
Novel set ranking			
3-10	3	3	2
11-30	2	2	2
31-100	1	2	3
101-300	1	1	2

When a group submitted in both rounds during the MouseFunc annotation prediction, the latest performance was used in the ranking. AUC, area under receiver operating characteristic curve; GO, Gene Ontology.

Our method performs the best relative to the other submissions on large terms, that is, terms with many annotations. In fact, two of our three first-place finishes are for the largest terms (biological process terms with 101 to 300 annotations and cellular component terms with 101 to 300 annotations). Conversely, two of our three third-place finishes are for the smallest terms (biological process terms with three to ten annotations and cellular component terms with three to ten annotations). Thus, our method consistently performs among the top methods used in this project across a range of functional categories, although it does appear that our best performance occurs on large and medium-sized terms. We will explore this trend in greater detail below in this section.

### Analysis of ensemble classifier performance

We further analyzed the individual components of our ensemble approach to gain knowledge about which methods perform the best under which circumstances.

#### Bagged SVM classifier performance is robust across a wide range of GO terms

**Figure 3:**
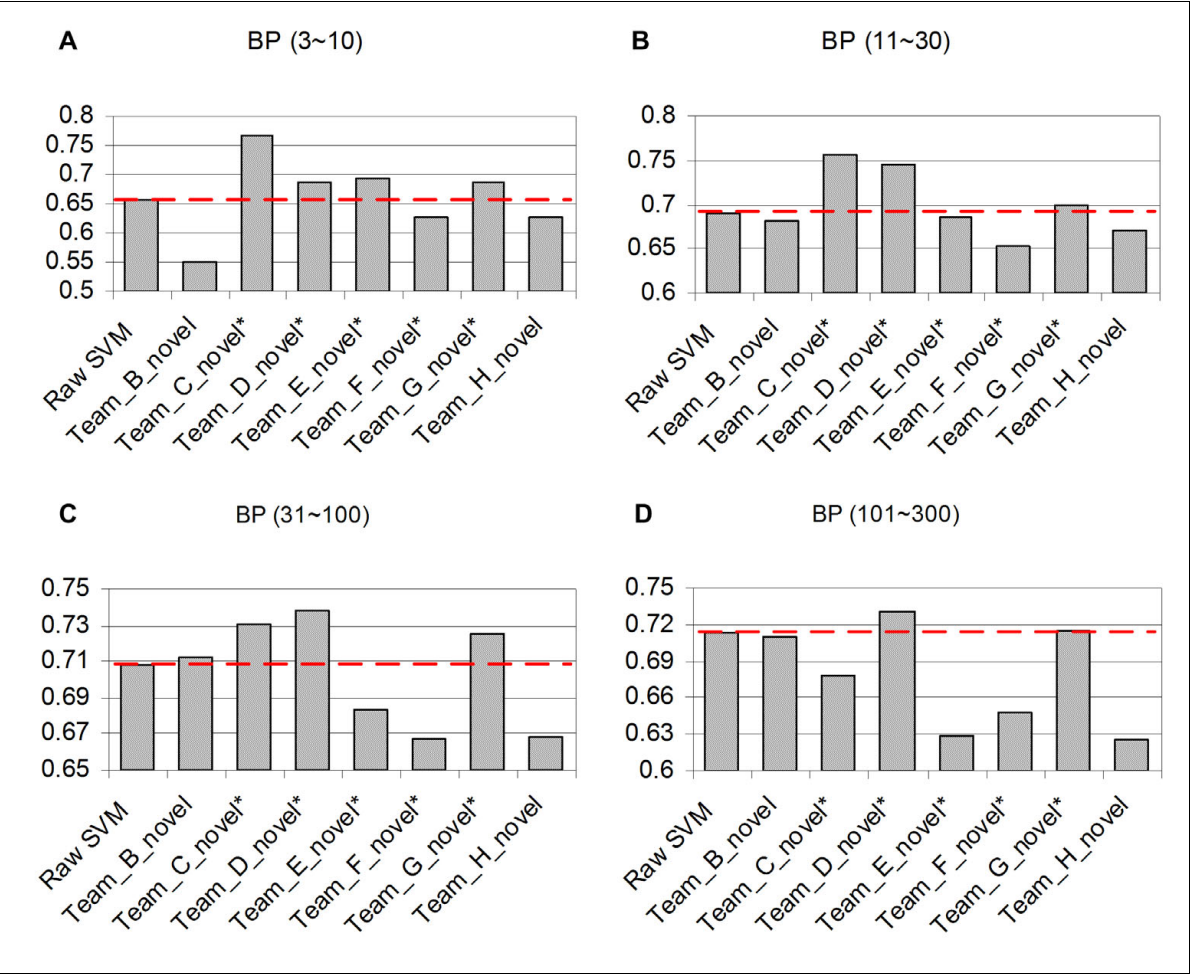
Comparison of the performance of a single SVM with other methods in predicting BP annotations of the novel set. This figure shows the AUC achieved by different submissions to the mouse function prediction project compared to a single SVM approach for GO BP terms of different sizes: **(a) **3 to 10; **(b) **11 to 30; **(c) **31 to 100; and **(d) **101 to 300. We found that a single SVM, after careful adjustment of parameters, performed relatively well across different sizes of GO terms, indicating the robustness of the SVM as a baseline prediction method. Only groups C, D and G performed better, on average, than the single SVM results. The dashed red lines represent the performance of single SVM in terms of AUC. Asterisks indicate second round submissions. AUC, area under receiver operating characteristic curve; BP, biological process; GO, Gene Ontology; SVM, support vector machine.

Since the SVM on a combined input dataset served as the basis for our approach, we first compared its performance alone on the evaluation set to submissions from other teams. The mean AUCs for biological process GO terms for the bagged SVM range from 0.63 (terms with 3 to 10 annotations) to 0.69 (terms with 31 to 100 annotations). With this performance, the linear-kernel SVM ranks consistently among the top three or four methods for all size categories of GO terms (Figure 3). In fact, on average, only three methods (groups C, D [our group] and G) performed better than the single SVM classifier across the entire set of biological process terms. Furthermore, only the ensemble approach described here consistently outperformed the single SVM predictions regardless of the GO term size, while group C performed significantly well on small terms. We were surprised at the relative success of this approach since our implementation was straightforward with little 'fine-tuning' with the exception of some input data processing and bootstrap aggregation (described in Materials and methods). However, this result confirms our original choice of the SVM as a robust baseline method for the more sophisticated classifiers in our ensemble.

#### Hierarchical correction of SVM classifiers improves performance

**Figure 4:**
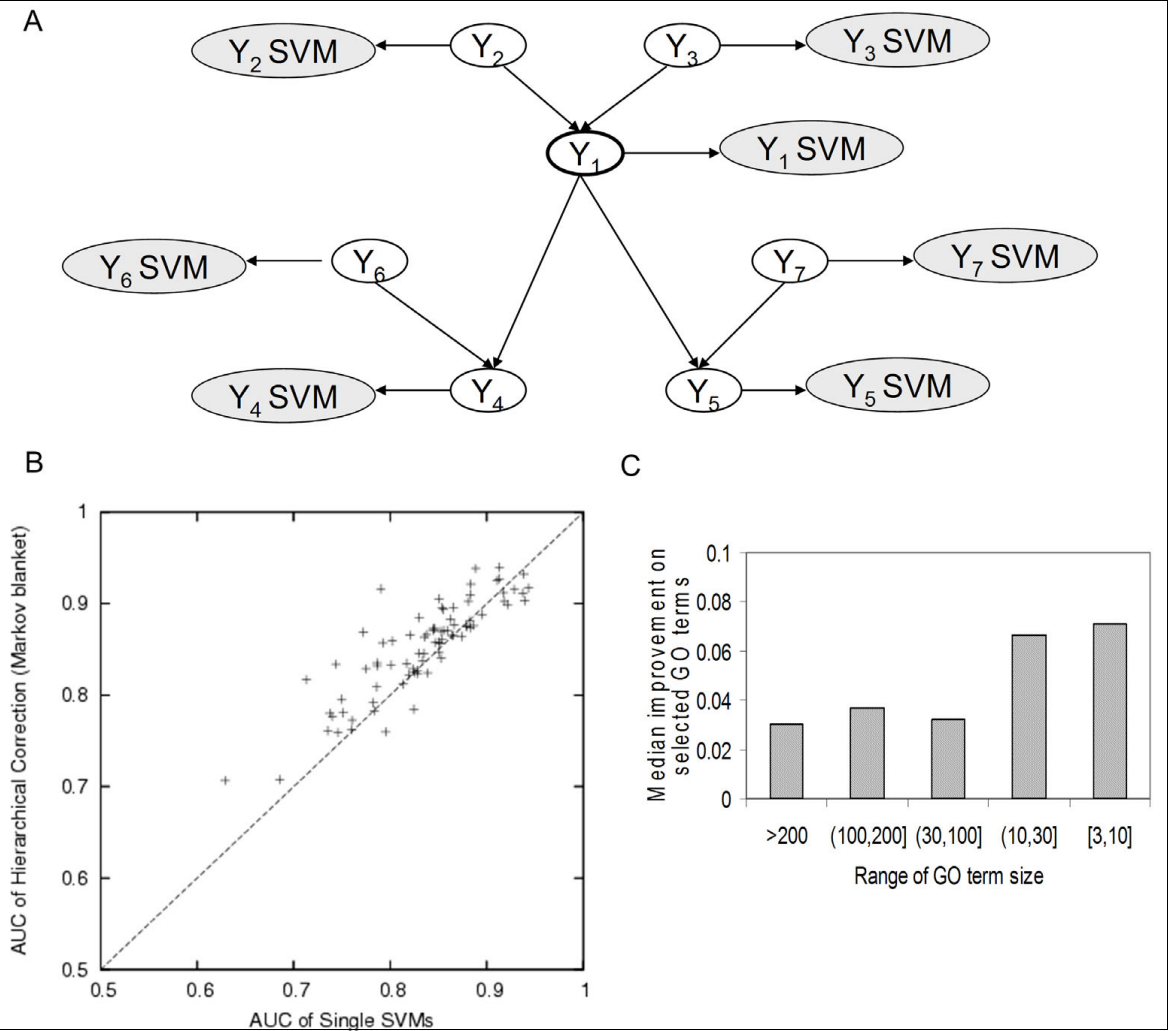
Hierarchical correction using Markov blanket structure. **(a) **Schematic of the local Markov blanket surrounding a GO term (Y1 is the node of interest in this example). Each GO term is represented by a blank node while the SVM classifier output for that GO node is represented by a shaded node. To address the hierarchical relationships between GO terms, for each GO term (Y1), we included all neighboring nodes in its Markov blanket to construct a Bayesian network. The distribution of SVM outputs (observed nodes) for positive and negative examples was encoded in the conditional probability tables of the Bayesian network. We then infer the probability of a particular gene's involvement in each GO term (a hidden node) based on its values in these observed nodes. **(b) **Improvement of the AUC for the novel set using the HIER-MB classifiers compared to single SVM predictions for selected terms for biological process terms of size 101 to 300 (number of genes annotated to this GO term in the training set). For each GO term, the best-performing sub-hierarchy was selected, and the ones that performed better than single SVM (characterized by held-out values in the training set) are plotted in this figure. **(c) **Median improvement of predictions for selected GO terms over different biological process GO term sizes. Hierarchical correction using Markov blanket structure performs better (when selected) for smaller terms. AUC, area under receiver operating characteristic curve; GO, Gene Ontology; HIER-MB, Markov blanket hierarchical correction; SVM, support vector machine.

**Figure 5:**
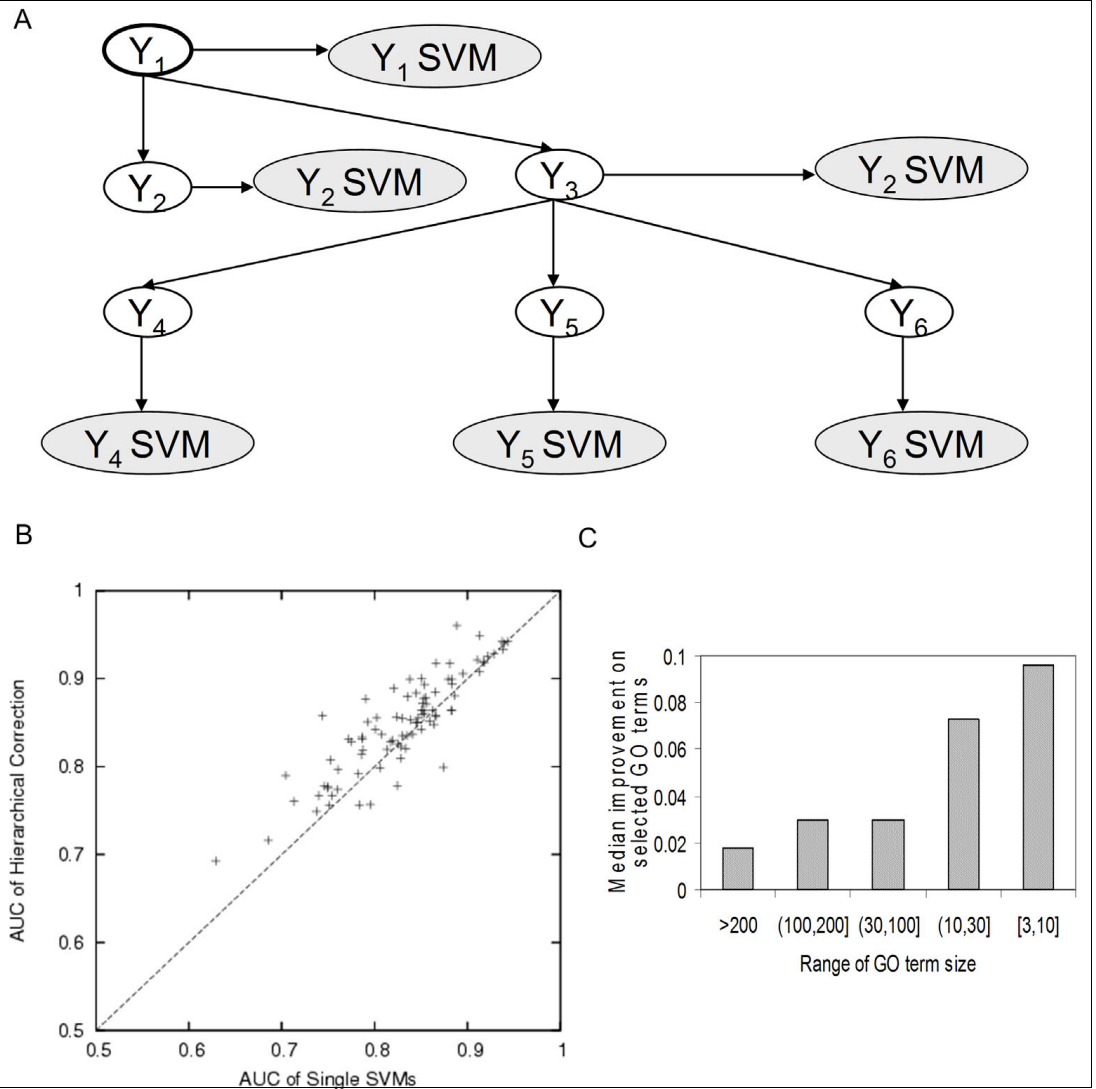
Hierarchical correction using BFS sub-networks. **(a) **Schematic of BFS sub-networks. Each GO term is represented by a blank node while the SVM classifier output for that GO node is represented by a shaded node. Starting from each GO term (Y1), we did a BFS to construct the local hierarchy, until a maximum of 30 terms were included. In this process, we considered only GO terms with five or more annotations in the training set. **(b) **Improvement of AUC for the novel set using the HIER-BFS classifier compared to single SVM predictions for selected terms for biological process terms of size 101 to 300 (genes annotated to this GO term in the training set). For each GO term, the best-performing sub-hierarchy was selected, and the ones that performed better than single SVM (characterized by held-out values in the training set) are plotted in this figure. **(c) **Median improvement of predictions for selected GO terms over different biological process GO term sizes. Again, hierarchical correction using BFS structure performs better for smaller terms when they were selected. AUC, area under receiver operating characteristic curve; BFS, breadth-first search; GO, Gene Ontology; HIER-BFS, breadth-first search hierarchical correction; SVM, support vector machine.

We further applied our hierarchical correction based on GO hierarchies and measured its improvement over single SVMs (see Materials and methods for details). For all GO terms with five or more annotations in the training set, we applied both approaches to subgraph generation, including the Markov blanket graphs (HIER-MB), and the breadth-first search graphs (HIER-BFS; Figures 4 and 5). We examined how often the hierarchical correction from either of these yielded better prediction accuracy than the bagged SVM predictions.

Predictions for a majority of GO terms are improved significantly by the hierarchical correction after selection of the subgraphs based on held-out values. The HIER-BFS classifier improved the single SVM predictions for 1,012 of 1,726 (59%) biological process GO terms by an average AUC of 0.049 (26% increase of random performance; Figure 5b). Similarly, the HIER-MB classifiers improved predictions for 996 of 1,726 (58%) terms by an average AUC of 0.044 (23% increase; Figure 4b). Through a combination of both strategies, the improvement is sustained across the whole range of GO term sizes, but both tend to improve prediction performance (AUC) more for smaller terms (Figures 4c and 5c). For instance, HIER-BFS improves predictions for terms with more than 200 annotations by an average of 0.018 (10% increase) but improves predictions for terms with between 3 and 10 annotations by an average of 0.095 (58% increase). We observed a similar trend for the HIER-MB classifier: 0.03 (17% increase) compared to 0.071 (43% increase) for smaller terms. Unsurprisingly, the two approaches improve predictions for largely the same set of GO terms. Of the 1,012 and 996 terms for which predictions were improved using the HIER-BFS and HIER-MB approaches, respectively, the combination of the two improved predictions for a total of 1,046 terms (61%) by an average of 0.048 (25%). Thus, including GO hierarchical information in function prediction can yield consistent improvements across a variety of terms, even in a challenging scenario such as mouse function prediction.

#### Naïve Bayes combination of SVMs on diverse data improves performance for large GO terms

**Figure 6:**
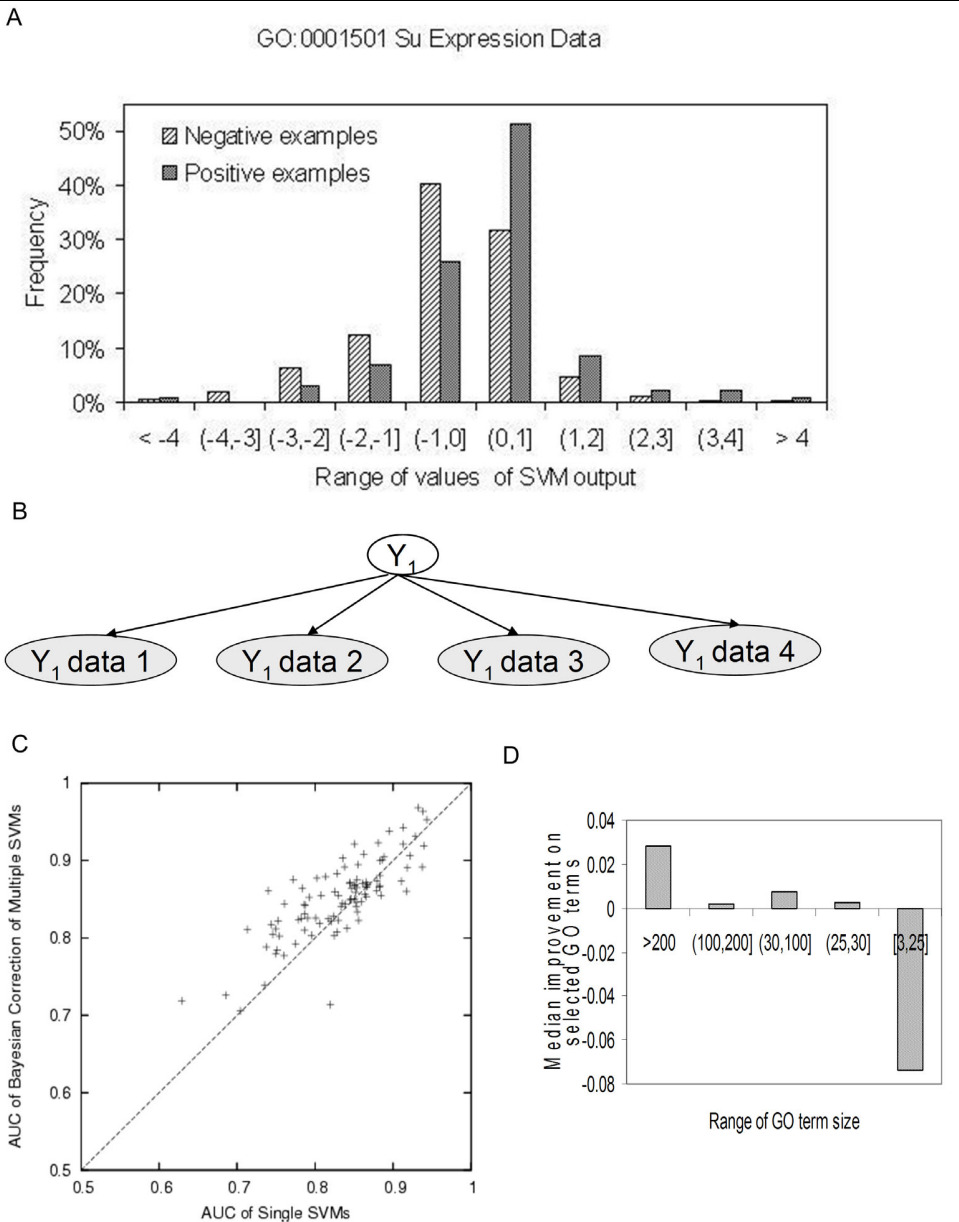
Bayesian combination of diverse datasets. **(a) **A typical example of distribution of SVM outputs of a single dataset. This distribution of SVM output (from the Su *et al. *expression dataset [34]) for positive examples is shifted slightly higher relative to the distribution of the negative examples. **(b) **Schematic of Bayesian combination of diverse datasets. For each GO term, we constructed a naïve Bayes classifier where the output of single-dataset SVMs was used as a single input node (observed node). **(c) **Improvement of AUC over single SVM predictions for selected terms for biological process terms with size 101 to 300 genes. The Bayesian combination of datasets was selected where the held-out results on the training set showed superior performance over the single SVM. **(d) **Median improvement of predictions for selected GO terms over different biological process GO term sizes. The Bayesian combination of diverse datasets performs well only for large GO terms. AUC, area under receiver operating characteristic curve; GO, Gene Ontology; SVM, support vector machine.

We also measured the improvement offered by the third classifier in our ensemble, the naïve Bayes combination of per-dataset SVMs. The motivation behind this approach is that genomic data are heterogeneous in the specific functions they capture and very different in their accuracy in predicting different functions, which may not be harnessed effectively by a single-kernel SVM classifier. For this approach, we trained an SVM on each individual dataset for each GO term, using bootstrapping to characterize the output distribution of positive and negative examples. Generally, the distribution of SVM outputs for held-out positive examples was shifted higher with respect to that of negative examples (for example, see Figure 6a). A naïve Bayes classifier on discretized SVM outputs was then used to assign a probability of functional assignment to a GO term of interest given such distributions from each dataset (Figure 6b).

A naïve Bayes combination of per-dataset SVMs results in a significant improvement over the single SVM classifier for several GO terms (Figure 6c). In all, it resulted in a significantly higher classification performance for 403 of 1,726 (23%) biological process GO terms. Interestingly, this set includes 87% of the terms with more than 20 annotations, suggesting this strategy is very effective for larger terms. Further analysis confirms this trend: the naïve Bayes classifier averages a 0.03 AUC improvement (17% increase) for terms greater than 200 annotations yet results in a loss of performance of 0.06 (36% decrease) on smaller terms. We suspect that this behavior is due to a lack of positive examples to characterize the per-dataset SVM output distribution, and in light of this observation, we restricted this method to terms with more than 20 annotations. This result demonstrates why an ensemble of different learning models is critical in predicting diverse gene functions: the naïve Bayes combination of single dataset classifiers works well for large terms but must be complemented with other approaches when few positive examples are available.

#### Analysis of the source of ensemble predictions

**Figure 7:**
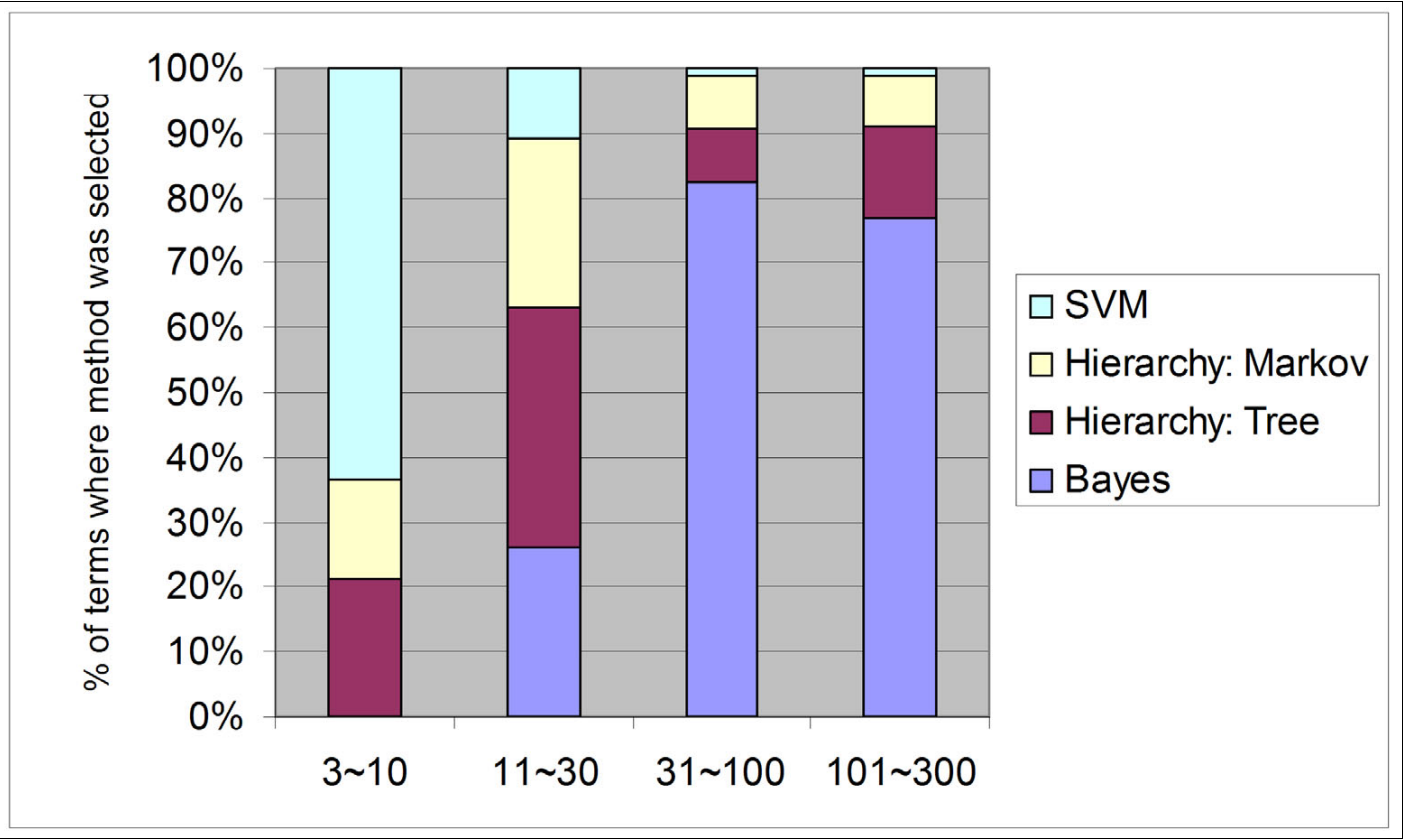
Composition of selected approaches for different GO term sizes. In our ensemble method, three different approaches based on SVM were applied, including bagged single SVMs, SVMs with hierarchical correction, and Bayesian integration of single-dataset SVMs. Each of them exhibits good performance in a different domain, which is indicated in this figure by the percentage of terms where each of the different methods exhibited the best performance for four different size categories. For smaller GO terms, the single SVM and hierarchical correction often achieve superior performance. For larger GO terms, Bayesian integration of diverse datasets performed better than the other two methods. GO, Gene Ontology; SVM, support vector machine.

Our ensemble method integrates output from the three base classifiers: the bagged single SVM, hierarchical Bayesian combination of SVM classifiers and the naïve Bayes combination of per-dataset SVMs. We evaluated how often each of these three approaches was selected for the final prediction set. As suggested by our earlier analysis, the majority of predictions are selected from the more sophisticated approaches, either the hierarchical classifier or the naïve Bayes combination (Figure 7). Specifically, the naïve Bayes combination was more likely to be selected for larger GO terms, while the hierarchical correction yields consistent improvements in all size categories, as suggested by our previous analysis. In all, for terms with more than 100 annotations, predictions for 99% were improved by one of these alternatives and predictions for 99%, 89%, and 37% were improved for GO terms with 31 to 100, 11 to 30, and 3 to 10 genes, respectively. Thus, the SVM provides a robust baseline classifier for gene function prediction, demonstrated by the performance on GO terms with fewer than five annotated genes, where only a single SVM was used. Nevertheless, SVM can often be significantly improved by leveraging unique properties of genomic data and the GO context, particularly when a reasonable number of positive examples are available.

### Discussion of evaluation results

We have demonstrated that our ensemble classifier achieves good performance across a variety of terms and performs consistently well relative to other methods contributed to the mouse function prediction project. As discussed earlier, one of the most important factors determining the relative performance of different methods was the size of the GO term (number of annotations or positive examples). The organizers grouped the set of predicted GO terms into 4 different size groups, based on the number of annotations in the training set: 3 to 10, 11 to 30, 31 to 100, and 101 to 300. These sets formed the basis of our earlier analysis. As one might suspect from the trends apparent in our method's performance, the participating groups' relative performance varied drastically across GO term size (Figure 2). For example, group B and group D (our group) were relatively better at predicting larger GO terms than smaller ones, while group C performed especially well on very small GO terms. We also observed that many groups, including groups E to H, performed well on intermediate-sized GO terms. These trends are usually consistent regardless of the GO term branch, indicating intrinsic properties of the specific methodologies used by each group.

The reasons for our improved performance at predicting larger terms are relatively clear based on our earlier analysis. First, the basis of our approach, the single SVM, shows significantly better performance than other approaches for larger GO terms. Second, as discussed above, the naïve Bayes combination is particularly effective on larger terms, which accounts for much of our superior performance in those cases. In general, because we used a supervised method for forming ensemble predictions (that is, through evaluation on the bootstrap held-out values), we require a reasonable number of examples to properly estimate relative performance of different classifiers. Thus, the ensemble yields improvement only where there are sufficient positive examples to support this estimation.

The variation among groups suggests an important lesson about gene function prediction. Namely, there is rarely one method that performs the best in all prediction scenarios. We anticipated this in designing our approach, which motivated our choice of an ensemble of classifiers, although all of our methods tended towards better performance on medium to large GO terms. Fortunately, there were other methods applied in this annotation prediction effort that show the opposite trend (for example, group C), suggesting that only a handful of methods may be able to provide superior results for most situations. In fact, a combination of our ensemble method's results for large terms and group C's results for small terms would nearly dominate all categories.

**Figure 8:**
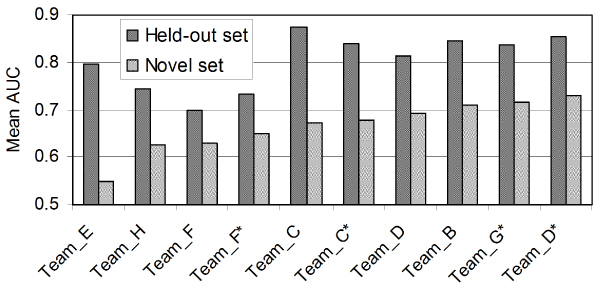
Relative performance of different methods with regard to the test set and novel set on GO biological process terms (size 101 to 300). The relative performance of individual groups differs between the test set and novel set. In addition, the performance on the novel set was generally worse than on the test set. This indicates that cross-validation should be used carefully in assessing the relative performance of different algorithms and that evaluation on novel biology is necessary. Asterisks indicate second round submissions. GO, Gene Ontology.

Another major source of variation in the relative performance of the different methods was the choice of the evaluation set. As mentioned earlier, two sets of annotations were used by the organizers to evaluate the submissions. The first evaluation was completed on a set of annotations simply held-out at the time of dataset distribution (the held-out set). Additionally, we were also able to evaluate our results on the new biological knowledge curated during the approximately eight month period after data assembly (the novel set). Our discussion here has been limited to evaluation of our method on the novel set, mainly because we think this set best models the real prediction scenario. Interestingly, we found that the relative performance of the different groups significantly differs between the held-out set and the novel set, although there is a slight correlation (Figure 8). Furthermore, the average AUC across all categories and groups drops significantly between the held-out set and the novel set. One characteristic that would potentially explain these differences is that the novel annotation set is dominated by ISS (inferred from sequence or structural similarity) annotations, while the test set has a significant fraction of RCA (inferred from reviewed computational analysis) annotations (Figure S3 in [9]). The similarity of the distributions of evidence codes between the test set and the training set makes the test set an easier prediction problem, which is confirmed by overall higher performance on that set. Despite these differences between the two evaluation sets, we were encouraged that our method performed among the top submissions using either benchmark (Table 1). In general, however, this alerts one to the fact that we should be very critical when evaluating our computational results using cross-validation analysis alone.

### Literature follow-up on novel mouse gene function predictions

Encouraged by the good performance of our method across a variety of biological processes, we investigated a number of specific predictions for previously uncharacterized mouse genes. For example, in predicting the genes involved in chromatin modification (GO:0016568), we achieved an AUC of 0.8396 in the novel set, and 80% precision at 20% recall. One gene of high confidence (0.99) but not annotated to this term was *PRDM14 *(MGI:3588194). This gene has no biological process annotations yet, and based on previous computational analysis, this gene is suggested to localize to the nucleus [10]. Our prediction of its involvement in the chromatin modification process correlates with the findings of a recent publication that showed that stable expression of PRDM14 up-regulated expression of a variety of genes involved in breast cancer [11]. In particular, PRDM14 contains a PR domain, possibly a derivative of SET, which is known to affect chromatin structure [12]. Oncogenic properties have been shown in other chromatin modifiers as well, including EZH2 and SMYD3 [13,14]. Given this evidence, it is likely that PRDM14 regulates specific gene expression levels through the modification of chromatin structure. This literature study suggests that using our approach can enhance the efficiency of novel gene function discovery, including the identification of genes involved in a core process such as chromatin modification, which is potentially relevant to our understanding of human biology.

### Experimental validation of gene function predictions in *S. cerevisiae*

The evaluations described above were based on cross-validation analysis and literature follow-up of specific predictions. We wanted to further verify that we are able to predict novel biology using our approach and, furthermore, that our method could be successfully extended to other species. Thus, we also applied our ensemble method (including single SVM, hierarchical correction and naïve Bayes integration of diverse datasets) to *S. cerevisiae*, and validated the top predictions of mitochondria-related function (GO:0007005, mitochondria organization and biogenesis). For this term, the naïve Bayes combination of diverse SVMs was selected as the best-performing method. The top uncharacterized gene predicted by our methods was *ICY1*. Though the function of this gene remains unknown, it was identified as a high-copy suppressor of the TIM22 mitochondrial protein import complex [15]. Furthermore, when *ICY1 *is deleted, these cells require mitochondrial DNA for survival (*Saccharomyces *normally can survive without mitochondrial DNA) [15]. Though the specific function of *ICY1 *remains unclear, these genetic phenotypes strongly suggest a role in mitochondrial membrane maintenance.

**Figure 9:**
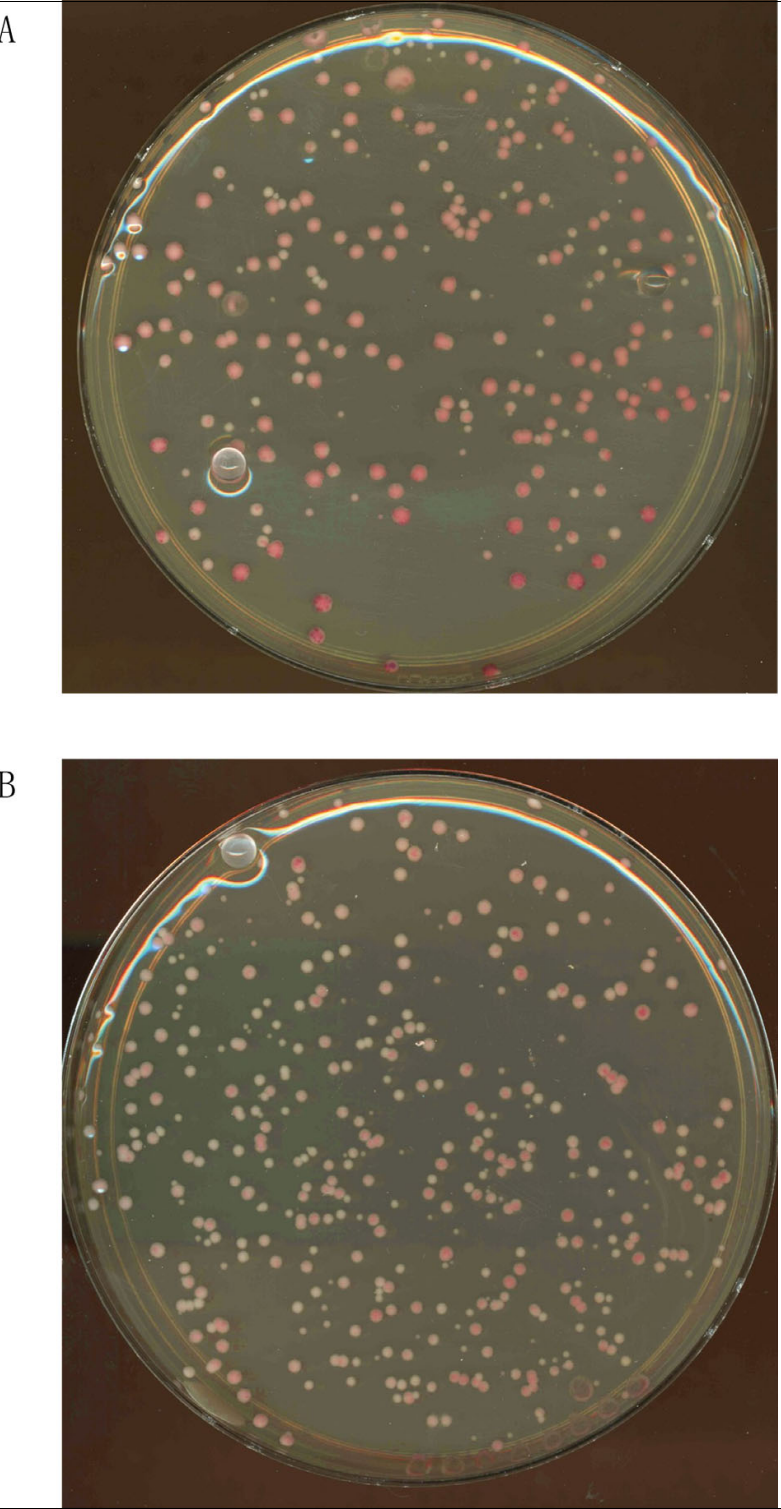
Petite assay on the *oms1Δg*utant. To further evaluate the performance of our ensemble algorithm, we applied our methodology to *S. cerevisiae*. We tested the second most significant uncharacterized gene with predicted involvement in mitochondria organization and biogenesis (GO:0007005). For this GO term, the Bayesian integration of single-dataset SVM was selected as the best performer in the ensemble. Using an assay for measuring the frequency of petite colonies for deletion mutants, we found that compared to **(a) **the wild type, **(b) **the *oms1Δ *mutant showed 67% more cells without functional mitochondria. GO, Gene Ontology; SVM, support vector machine.

The second top uncharacterized gene was *YDR316W *(*OMS1*). Similar to *ICY1*, *OMS1 *was identified as a high copy suppressor of a mitochondrial protein (Oxa1). Though localization studies [16,17] have identified Oms1 as a mitochondrial protein, its function remains a mystery. Using a classic assay to measure perturbations in normal mitochondrial function [18], we experimentally verified that Oms1 is required to maintain functioning mitochondria. Briefly, wild-type and *oms1Δ *strains were grown in glycerol, which selects for cells that have functional mitochondria. These cultures are then plated for single colonies on rich media, which allows growth of cells without functional mitochondria. Resultant colonies are grown on rich media to measure the proportion of cells in the parent colony that lost mitochondrial function. This is done by plating single cells from the colony on rich media and overlaying the resulting colonies with tetrazolium, which stains colonies that have functioning mitochondria red while colonies lacking functioning mitochondria stay white (Figure 9). Eight independent frequencies were measured for both *oms1Δ *cells and wild type. The *oms1Δ *cells had 67% more colonies without functional mitochondria compared to wild type (*P *< 0.003, Mann Whitney U-test). This assay clearly demonstrates that Oms1 is required for the normal production of functional mitochondria and provides a strong validation of our prediction methodology.

## Conclusion

We have described an ensemble approach to gene function prediction based on three configurations of SVM classifiers. Specifically, we have demonstrated how the hierarchical context of the GO can be used to refine independent GO term predictions and how a naïve Bayes combination of single dataset classifiers can harness diverse functional information in heterogeneous data. We have shown that an ensemble of these approaches can significantly improve GO term predictions across a range of processes and performs very well relative to a number of other state-of-the-art machine learning approaches. Finally, we have demonstrated through prediction of novel annotations for mouse and experimental validation of our predictions in yeast that this method can be used to precisely discover new biology.

## Materials and methods

### Bagged SVM classifier for each GO term

The basis of our approach is a SVM classifier, which is a state-of-the-art machine learning method that has been used successfully for supervised learning on high-dimensional data in a number of different application domains [19]. Our application here is straightforward: we combine the raw input data into a single dataset, train a linear-kernel SVM on each GO term of interest using annotated genes as positive examples, and use bootstrap aggregation (bagging) to derive predictions. We evaluated several variations on data pre-processing and bootstrapping, and highlight some of the critical factors here.

One of the most critical choices in applying the single SVM was the way in which features from diverse input data types were combined. We found that direct concatenation of all datasets led to over-weighting of datasets with more features (for example, protein-protein interaction data), which is consistent with previous reports of feature scaling for improved performance (for example, [20]). Thus, to normalize each dataset's contribution to the Gram matrix, we separated all feature vectors for each gene into dataset-specific subsets of components, that is:

$${\vec{v}}_{i}=\left[{\vec{v}}_{i}^{1},{\vec{v}}_{i}^{2},\cdots ,{\vec{v}}_{i}^{k}\right]$$

where ${\vec{v}}_{i}^{k}$ is the feature vector for gene *i *from dataset *k*. Each of these dataset-specific gene vectors were then normalized separately as follows:

$${\vec{v}}_{i}^{k'}=\frac{{\vec{v}}_{i}^{k}}{\left\|{\vec{v}}_{i}^{k}\right\|}.$$

All normalized features for each gene were then concatenated in a single input matrix, *D*:

$$D=\left[\begin{array}{cccc} {\vec{v}}_{1}^{1'} & {\vec{v}}_{1}^{2'} & \ldots & {\vec{v}}_{1}^{k'}\\ {\vec{v}}_{2}^{1'} & {\vec{v}}_{2}^{2'} & \ldots & {\vec{v}}_{2}^{k'}\\ \vdots & \vdots & \ddots & {\vec{v}}_{n}^{k'} \end{array}\right].$$

This matrix, *D*, defined the set of features for each gene that were input into each SVM. This simple procedure dramatically and consistently improves classifier performance. Before this normalization, all input genomic datasets were pre-processed as described in the 'Implementation details' section below.

A second critical aspect of our application of single SVMs was our use of bootstrapping. We applied bootstrap aggregation (bagging) for each GO term, where examples (genes) were randomly sampled with replacement (0.632 bootstrap, that is, the expected fraction of selected data points is 0.632) [21]. For each bootstrap sample, a model was learned based on the selected examples, and the resulting classifier was used to give an output for both non-selected (out-of-bag) examples and the unknown examples. The final classifier outputs were taken as the median of out-of-bag values across bootstraps for the training set, and as the median of all values across bootstraps for the unknown examples. We found that performance rises sharply with the number of bootstrapping rounds and levels off after approximately 25 rounds for all GO terms.

### Bayesian hierarchical combination of SVM classifiers

The second component of our approach is a method for refining independent GO term classifiers by integrating them in the context of the GO hierarchy. The method we propose here is an extension of a simpler approach we developed and successfully applied to the problem of yeast gene function prediction in earlier work [22].

To briefly summarize, the basis of the approach is a Bayesian network that enforces hierarchical consistency across GO terms (for example, if a child node is predicted positive, then the parent node must also be a positive prediction). Each hidden node in the Bayesian network corresponds to a GO term and is associated with an observed node, which is the output of the single SVM for that GO term (Figures 4a and 5a). The Bayesian framework encodes both the hierarchical rules and the classification performance of the single node classifiers [22]. In our formulation here, the SVM classifiers at each node are first trained on the GO terms independently and performance characteristics are derived from bootstrapped outputs on held-out genes.

We apply this approach as described in [22], but extend it to work for the entire GO hierarchy (the initial method was developed for a small set of GO terms). To make inference on our Bayesian networks feasible, we split each GO branch (biological process, molecular function and cellular component) into several smaller subgraphs, each preserving the local neighborhood around each term. We used two different approaches for defining these subgraphs.

First, for each GO node on which we made predictions, we constructed Bayesian networks based on subgraphs including only other GO terms in its Markov blanket (Figure 4a). The nodes in a Markov blanket for Y are typically a subset of its parents, its children and its children's parents. The values of the variables corresponding to the nodes in the Markov blanket for Y contain all the necessary data to make a prediction for Y [23]. We refer to this approach as HIER-MB. Our second approach for defining subgraphs was to apply a breadth-first search (BFS) from each GO term to recover all descendants up to a maximum of 30 total GO terms, restricting our set to only terms with ≥ 5 annotations in the training set (Figure 5a). GO term predictions were made for all nodes present in these subgraphs. We refer to this method as HIER-BFS. Using these two approaches, each GO term occurred in several Bayesian networks, reflecting slightly different local GO term contexts. Finally, hierarchically corrected predictions for each GO term were chosen on the basis of the performance of the median of bootstrap outputs for bootstrap held-out genes.

### Naïve Bayes combination of SVM classifiers on diverse data

The final component of our ensemble is a naïve Bayes combination of per-dataset SVM classifiers. The motivation behind this approach is the understanding that a single-kernel SVM on a concatenation of several diverse datasets may be less than optimal for the heterogeneity of the input genomic data. Thus, we applied an approach that learns at a dataset-specific level, but that also robustly integrates across several datasets. A naïve Bayes combination of per-dataset SVMs is ideal for this setting; naïve Bayes classifiers have been widely applied in a variety of domains and are known for their robust performance on diverse biological settings [5] and SVMs are able to provide good classification performance, particularly on high-dimensional datasets. This combination enabled us to capture the heterogeneity across datasets while still harnessing the accuracy of the SVM. SVMs were trained independently on each of the input datasets, and these outputs were combined with a naïve Bayes classifier on the basis of the held-out set performance. We used linear kernel SVMs for all datasets except the protein-protein interaction data, where we used a diffusion kernel [24] because it showed superior performance. Previous work on integration of diverse data with SVMs has focused on kernel-level integration (for example, [25-27]). We suggest this as an alternative, with the possible advantage that our framework allows for non-linear combinations of the input data, which is not always the case for kernel-level integration methods. Although direct comparison of these two approaches is beyond the scope of this work, we find that the one presented here can significantly improve a single SVM classifier's predictions in many cases (see Results). For all dataset classifiers, we used bootstrap aggregation (bagging) over 25 bootstrap samples, reporting the median output of all held-out SVM instances for each gene. These held-out values were used to characterize the distribution of positive and negative examples for each dataset and GO term (Figure 6a). We then applied a naïve Bayes classifier to estimate the probability of annotation to a certain GO term, given SVM outputs of all datasets for a gene:

$$P(GOY|E_{1},E_{2},...,E_{n})=\frac{1}{Z}P(GOY)\prod_{i=1}^{n}P(E_{i}|GOY)$$

where GOY stands for a positive GO term annotation (Yes), *E*_*i *_represents the value of the SVM output for dataset *i*, and *Z *is a scaling factor depending only on *E*_1_, *E*_2_,.... *E*_*n*_.

### Forming an ensemble prediction

We measured the performance of each classifier (single SVM, hierarchical correction of SVMs, and a naïve Bayes combination of SVMs on diverse datasets) on each term using the AUC for the performance of the median of out-of-bag bootstrap outputs and selected the best-performing approach for each GO term.

One could imagine a more sophisticated scheme for combining these approaches and, in fact, we experimented with several. For instance, we evaluated using the naïve Bayes combined predictions as input to the hierarchical correction network. However, this and several other more complex methods resulted in poor classification performance, often characterized by overfitting. As we illustrate in the Results section, each of our individual methods shows improvement in relatively distinct areas of the GO, which may explain why simple combination schemes are effective.

### Implementation details

#### Pre-processing of functional genomic data and gold standard

The MouseFunc benchmark datasets used in this study were provided by the organizers [9] and consisted of both binary and continuous features. The binary datasets, often very sparse, included the InterPro domain data [28], MGI phenotype data [29], Online Mendelian Inheritance in Man (OMIM) disease data [30] mapped to laboratory mouse [31], PfamA domain data [32] and protein-protein interaction data [33]. Because only non-zero entries were used as SVM input, feature columns that contained less than three non-zero entries were removed from the data matrix. All missing data were treated as zero entries.

Continuous-valued features included the Su *et al. *expression data [34], Zhang *et al. *expression data [35], expression data from Serial Analysis of Gene Expression (SAGE), and phylogenetic profiles from InParanoid [31]. All entries in these data matrices are usually non-zero. However, the values vary in scale across data matrices, making direct application of the SVM classifier impractical. As a result, for each continuous dataset, we z-score normalized the data by feature to zero-mean and unit-variance before normalizing each dataset's contribution to the Gram matrix (see the 'Bagged SVM classifier for each GO term' section).

All methods used here for gene function prediction are supervised, and thus require a gold standard set of positive and negative examples for each GO term. The positive examples for each term were taken as genes annotated directly or to a descendent term. Negative examples were assumed to be all other genes in the training set.

#### SVM implementation and parameter selection

In this study, we used the SVM^light ^software [36] as a basis to implement all SVM classifiers. We experimented with several parameters, including *C *(trade-off between training errors and margin), *w *(epsilon width), and *j *(cost-factor) over a wide range. We found that only the cost-factor had a significant impact on the classifier performance on our data, and we set its value to the ratio of negative examples to positive examples in the training set. With the exception of a diffusion kernel on the protein-protein interaction data, all other SVMs used a linear kernel. We also experimented with a radial basis function kernel but found that it resulted in poorer performance for many GO terms. For Bayesian network inference, we used the University of Pittsburgh Decision System Laboratory's SMILE library and GENIE modeling environment [37].

#### Computational cost of the algorithm

The most computationally intensive part of our method is training the SVMs, which in the worst case, is linear in the number of training examples (*n*) and the number of non-zero features (*s*) (*O*(*sn*)) [38]. SVMs are trained for each GO term, each dataset (approximately 10), and each bootstrap fold (25 total). The structure of the Bayesian networks for hierarchical correction are fixed based on the structure of the GO and the size of each subgraph is limited such that the time complexity of inference on each network is minimal (fewer than 30 total nodes). Parameter estimation for both the hierarchical and naïve Bayes classifiers is based on out-of-bag bootstrap values from the SVM classifiers and is also minimal compared to the SVM training phase.

### Experimental protocol for yeast mitochondria assay

Yeast is able to grow and proliferate even without functional mitochondria on fermentable carbon sources. As such, yeast cells occasionally fail to pass respiratory competent mitochondria on to daughter cells, but these cells can continue to proliferate. Cells lacking functional mitochondria are called petite cells. In this assay we assessed the rate at which single gene knockout strains produced petite offspring as adapted from [18].

The *oms1Δ:kanMX *strain was obtained by fresh sporulation of the heterozygous deletion strain from the international consortium collection [39]; six independent spores were isolated by single colony purification on selective media for the kanMX cassette.

For each mutant strain tested, we grew several replicates of the strain for 48 hours in liquid YP Gycerol at 30°C [40]. Strains able to grow on glycerol were diluted and plated for single colonies on YPD plates, which releases the requirement for functional mitochondria. Thus, as colonies formed, cells without functional mitochondria were generated. When the colony is fully formed it comprises a mixture of cells with functional mitochondria and cells without functional mitochondria. We measured this ratio by re-suspending a colony and plating a dilution of this re-suspension such that 100 to 300 colonies were formed on a YPD plate. By overlaying with soft agar containing tetrazolium, cells with functional mitochondria were stained red, while cells without functional mitochondria remained white. The final mixture for agar overlay contained 0.2% 2,3,5-triphenyltetrazolium chloride (Sigma, St Louis, MO, USA), 0.067 M phosphate buffer pH 7.0 and 1.5% bacto agar. The ratio of white cells to total cells gives the petite frequency. Eight independent petite frequencies (biological replicates) were measured for each strain tested. The distribution of these frequencies was compared to the frequency of petite generation in wild-type yeast.

## Abbreviations

AUC, area under ROC curve; BFS, breadth-first search; GO, Gene Ontology; HIER-BFS, breadth-first search hierarchical correction; HIER-MB, Markov blanket hierarchical correction; MGI, Mouse Genome Informatics; ROC, receiver operating characteristic; SVM, support vector machine.

## Competing interests

The authors declare that they have no competing interests.

## Authors' contributions

YG was responsible for developing, implementing and applying the approach and drafted the manuscript. CLM assisted in developing and applying the method, helped in interpreting the results, and helped draft the manuscript. DH and AC performed experimental confirmation on the yeast deletion mutant, and DH helped draft the manuscript. ZB originally conceived the idea of hierarchical correction and provided guidance in applying it to mouse data. OGT supervised the effort and provided feedback throughout the process. All authors read and approved the final version of the manuscript.
